# Effective surgical treatment of life-threatening huge vascular anomalies associated with thrombocytopenia and coagulopathy in infants unresponsive to drug therapy

**DOI:** 10.1186/s12887-020-02093-x

**Published:** 2020-04-27

**Authors:** Yaohao Wu, Ronglin Qiu, Lexiang Zeng, Liyang Liang, Jie Zhang, Jiajia Zhou, Wenli Jiang, Jianhang Su, Xiaogeng Deng

**Affiliations:** 1 Department of Pediatric Surgery, The Sun Yat-Sen Memorial Hospital of Sun Yat-Sen University, No. 107 Yanjiang West Road, Guangzhou, 510120 People’s Republic of China; 2 Department of Pediatric, The Sun Yat-Sen Memorial Hospital of Sun Yat-Sen University, Guangzhou, China

**Keywords:** Infant, Vascular anomalies, Kasabach-Merritt phenomenon, Surgical treatment

## Abstract

**Background:**

Systemic drug therapy is generally recommended for infant huge vascular anomalies associated with thrombocytopenia and coagulopathy, but some patients are not suitable due to drug unresponsiveness or life threatening conditions before the drug works, who will need to receive surgical treatment. This study retrospectively analyzed the clinical features, imaging features, and surgical outcomes of these patients.

**Methods:**

The clinical data of 4 infants with huge vascular anomalies (2 vein malformations (VMs) and 2 kaposiform hemangioendothelioma (KHE)) associated with thrombocytopenia and coagulopathy treated from June 2016 to December 2017 were retrospectively analyzed. All patients received glucocorticoids, propranolol, vincristine or sirolimus treatment before admission, but the treatment was ineffective. Skin petechia, thrombocytopenia and coagulopathy were present at the time of admission. CT scanning was performed before operation. The patient’s general clinical data, hematological examination results, operation time, surgical bleeding volume, blood transfusion volume and surgical complications were collected for analysis. The patients were followed up for 10–26 months.

**Results:**

CT scanning results of 2 patients showed special CT features without detectable enhancement within the lesion after CT enhanced scanning and multiple phleboliths formation. Four patients underwent surgical treatment successfully. Two patients underwent complete resection of the lesion, and 2 underwent cytoreductive surgery. Preoperative clinical symptoms such as skin petechia, thrombocytopenia and coagulopathy were normal at 1 week after surgery. Postoperative pathological results showed 2 cases of KHE and 2 cases of VMs. All patients were discharged from hospital without physical dysfunction, recurrence, or death.

**Conclusions:**

Timely and appropriate surgical intervention can achieve satisfactory results for infants with huge VMs and KHE who were unresponsive to drug therapy or suffering from life-threatening occasion before the drug become effective.

## Background

Huge vein malformations (VMs) with or without arteriovenous malformation can be associated with thrombocytopenia and coagulopathy. Sarkar et al. first reported the Kasabach-Merritt phenomenon (Kasabach-Merritt phenomenon, KMP) in 1997. KMP is defined as kaposiform hemangioendothelioma (KHE) or tufted angioma associated with profound thrombocytopenia, together with consumptive coagulopathy and hypofibrinogenemia. KHE with KMP should be differentiated from the clotting disorder associated with extensive VMs. In patients with extensive VMs, especially involving the trunk and/or extremities, localized intravascular coagulopathy can occur at baseline and worsen by any aggravation of the malformation such as trauma or surgery [1]. The levels of fibrinogen are low, and associated with elevated D-dimer and fibrin degradation products. However, the thrombocytopenia is less profound in VMs than in KHE with KMP [2]. Patients often die from coagulopathy, vascular malformation rupture, gastrointestinal bleeding, intracranial hemorrhage, sepsis, and damage to vital organs, with mortality rates as high as 10 to 37% [3] [4] [5].

Drolet et al. reported the consensus derived practice standards plan for the treatment of complicated KHE in 2013 [6]. Ji et al. demonstrated satisfactory efficacy of sirolimus with a reasonable safety profile in patients with progressive KHE [7]. Many Therapeutic effects were achieved by systemic corticosteroids, propranolol, interferon, sirolimus or chemotherapy treatment [8–15] Sirolimus is used as first-line drug for the treatment of KMP [1] [7] [15]. However, the effectiveness of single or multi-drug combination therapy is still unsatisfactory due to long treatment time, side effects, disease recurrence and death [16]. Some patients are unresponsive to drug treatment, resistant to drug treatment, or have life threatening symptoms before the drug works [12] [17]. As a result, management of huge KHE with KMP or VMs combined with localized intravascular coagulopathy remains challenging to date. In the case of ineffective drug treatment, surgery may be the only way to save patients, even though surgery itself carries high risk and complete removal of the vascular tumor or VMs is difficult. In this study, we retrospectively analyzed the clinical characteristics, imaging features, and surgical outcomes of infants with huge KHE and KMP or VMs combined with localized intravascular coagulopathy, who were unresponsive to drug treatment or developed life-threatening conditions during drug treatment.

## Methods

The clinical data of 4 infants admitted for surgical treatment of huge vascular anomalies (2 VMs and 2 KHE) combined with thrombocytopenia and coagulopathy from June 2016 to December 2017 were retrospectively analyzed. Before the operation, four patients were treated with glucocorticoids, glucocorticoids combined with vincristine, glucocorticoids and propranolol combined with sirolimus in different hospitals, and the treatment was ineffective. Systemic skin ecchymosis, severe thrombocytopenia (platelet count range 10–19 × 10^9^/L) and coagulopathy (fibrinogen less than 1 g/L, PT, APTT, and PTINR cannot be detected) were presented in the children at the time of admission. After admission, all patients underwent CTA scanning before surgery in order to visualize the location of lesion invasion, lesion size, lesion composition and its relative location with major blood vessels. The children’s platelet count, PT, APTT, PTINR, fibrinogen, and D-dimer were monitored. The patients also received an infusion of fresh frozen plasma and platelets to improve platelet function and blood coagulation. Patients who were unresponsive to drug treatment or who had developed systemic skin ecchymosis, platelet count less than 20 × 10^9^/L, and concurrent coagulopathy (such as fibrinogen less than 1 g/L, PT, APTT, and PTINR cannot be detected, etc.) were considered for surgical treatment. On the day of surgery, the patients started surgery immediately after receiving fresh frozen plasma, fibrinogen, and platelet transfusion. The patient’s general clinical data, hematological examination results, operation time, surgical bleeding volume, blood transfusion volume and surgical complications were collected for analysis. All patients were followed up for 10–26 monthsafter surgery, with an average period of 18.75 months.

## Results

**Fig. 1:**
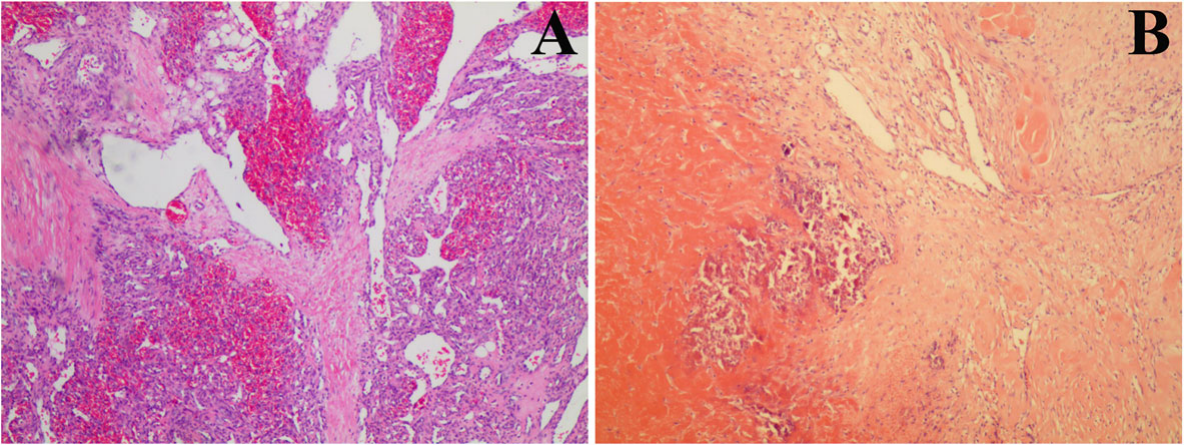
**a**: A large number of spindle-shaped endothelial cells aggregated in the dermis to subcutaneous fat layer, clustered into small lumens, and a large number of red blood cells were stagnated. **b**: There were blood sinuses with different sizes and shapes in the tissues, some of the blood sinuses had blood cell stasis, and some were thrombosis with a lot of calcification. The sinus wall was thin and uneven in thickness and contains the intima and media. Sinus were lined with a single layer of flat endothelial cells. The smooth muscle layer was close to the endothelial layer, and the muscle layer was thin. Hyperplasia of collagen fibers and granulation tissue

The demographic data of patients was shown in Table 1.There were 2 males and 2 females, aged between 1 and 11 months, with an average of 5.5 months. Among all four patients who received operation, the lesion found in cases 2 and 3 (see Table 1) were removed completely, and the remaining 2 cases underwent cytoreductive surgery. Postoperative pathological examination showed that case 1 and case 2 were VMs. The clinical manifestations of 2 patients were presented as a well-defined, cystic, non-inflammatory mass on the left back or right retroperitoneum. The skin color of the mass was not different from that of the surrounding, but was complicated with systemic skin ecchymosis. There was no dysfunction caused by mass compression in 2 cases. Microscopic examination showed that there were blood sinuses with different sizes and shapes in the tissues. Some blood sinuses had blood cell stasis, and some were thrombosis with a lot of calcification. The sinus wall was thin and uneven in thickness and contained the intima and media. Sinus were lined with a single layer of flat endothelial cells. The smooth muscle layer was close to the endothelial layer, and the muscle layer was thin. Hyperplasia of collagen fibers and granulation tissue (see Fig. 1b). Howerver, the pathological examination showed that case 3 and case 4 were KHE. The clinical manifestations of 2 patients were presented as a solitary tumor with red-purple, indurated, pebbly texture and ill-defined margins, and were also complicated with lower limb swollen, dysfunction, and systemic skin ecchymosis. Microscopy examination showed that a large number of spindle-shaped endothelial cells aggregated in the dermis to subcutaneous fat layer, clustered into small lumens, and a large number of red blood cells were stagnated (see Fig. 1a).

**Table 1:** Demographic data

Case	Age (M)	Weight (Kg)	Lesion location	Surgery	Pathology	Complications
1	4	7	thoracic cavity and thoracic vertebrae from the left back through the intercostal space	cytoreductive surgery	vein malformation	N
2	11	9	the right retroperitoneum	completely resection surgery	vein malformation	N
3	1	5	the right thigh	completely resection surgery	kaposiform haemangioendothelioma	N
4	6	9.4	the left pelvis, the lumbar and sacral coccygeal vertebra	cytoreductive surgery	kaposiform haemangioendothelioma	N

*M* Month; *Kg*: Kilogram; *N* None

**Fig. 2:**
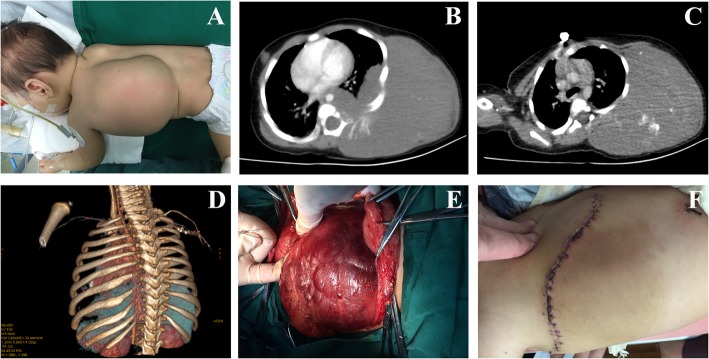
**a**: A huge mass can be seen on the left side of the back, about 23 cm × 15 cm in size. The mass is cystic and has a sense of undulation, but there is no pulsation. The mass is located under the skin and the skin color has no obvious change. **b**: CT scan showed a huge lesion from the chest wall invaded into the left thoracic cavity and thoracic vertebra through the intercostal space. Contrast-enhanced CT scan showed no obvious enhancement within the lesion. C: CT scan showed multiple phleboliths formation in the lesion. D: The three-dimensional reconstruction of CTA only found a small amount of blood-supplying vessels on the left side of the spine, and the whole lesion morphology was invisible. E: In the operation, the mass was identified as VMs with abundant blood supply and thin wall. When surgically separated, the mass was prone to rupture and massive bleeding. F: After the operation, the blood supplying of the flap was good, and the wound healed well

**Fig. 3:**
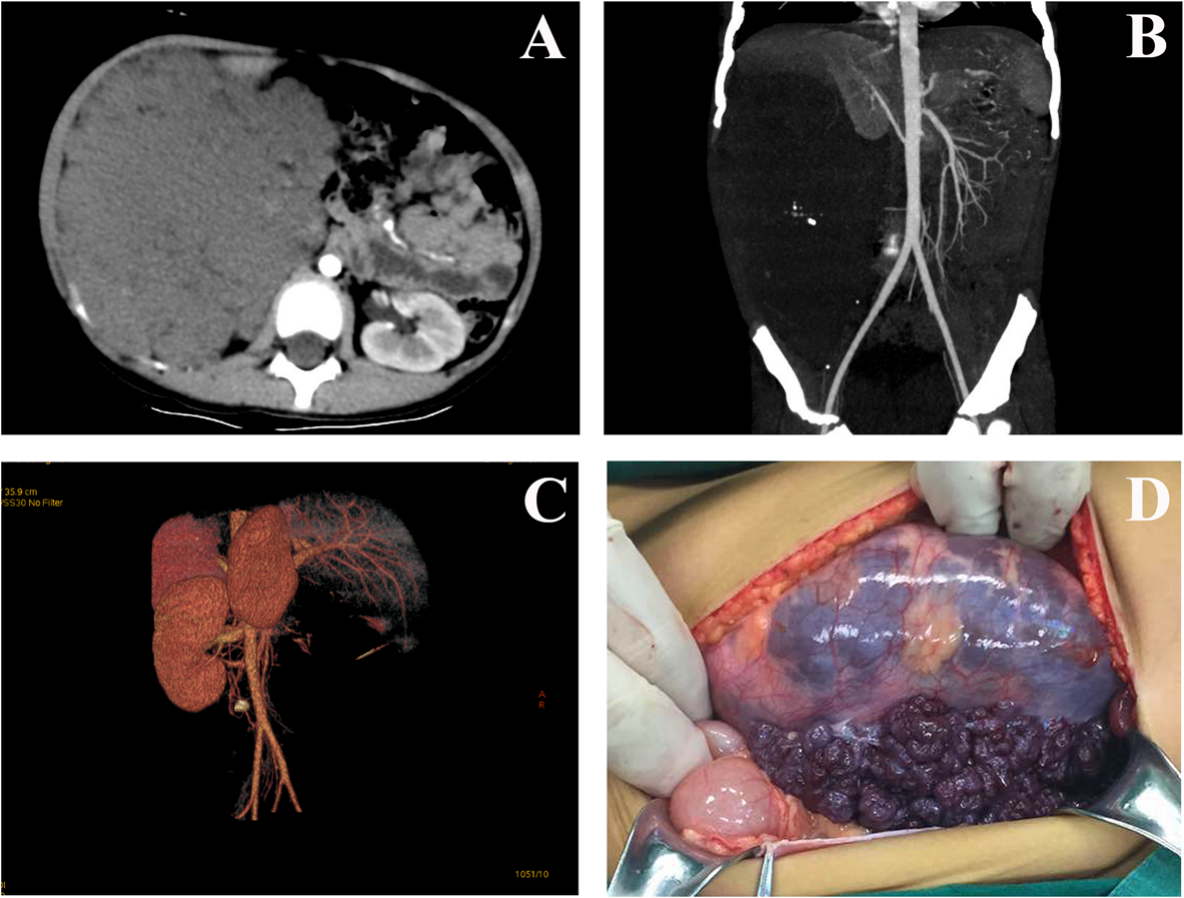
**a**: CT scan showed a large soft tissue mass in the right retroperitoneum, about 10 cm × 13 cm × 17 cm in size, and contrast-enhanced CT scan showed no obvious enhancement inside the mass. **b**: CT scan showed multiple phleboliths formed in the lesion, and the blood supplying vessels was invisible in the mass. **c**: Three-dimensional reconstruction of CTA did not show image of the right retroperitoneal mass, and the blood supplying in mass was not seen, either. **d**: In the operation, the giant soft tissue mass in the right peritoneum was venous malformation. The color of the mass was dark purple. Lesion blood vessels were flexion and expansion. The mass capsule was very thin and was prone to rupture and bleeding

**Fig. 4:**
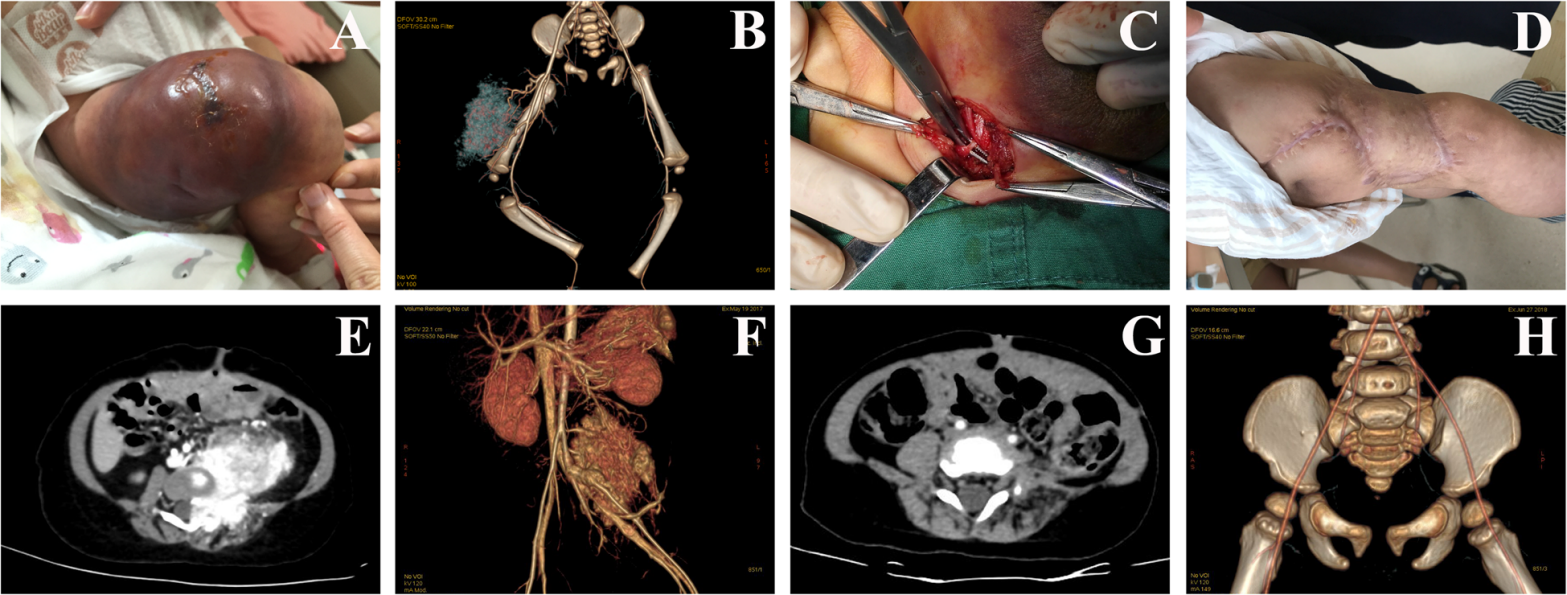
**a**: 1 month old baby’s right thigh with a huge KHE and KMP, the tumor occupied the outer side of the thigh. **b**: Three-dimensional reconstruction of CTA showed the tumor was feeding by the right deep femoral artery and its branches. **c**: In the operation, a part of the tumor feeding artery was ligated to reduce intraoperative bleedingat first. **d**: 1 year after tumor resection and skin grafting, the patient’s wound healed well and no recurrence was seen. **e**: Pelvic cavity CT scan of a 9-month-old baby showed a soft tissue mass in the left pelvic cavity. The boundary between the mass and the left psoas muscle and the iliopsoas muscle was unclear. The spinal canal was invaded, and the left side of L3–5 vertebrae structure was destroyed. Contrast-enhanced CT scan showed significant enhancement within the tumor. **f**: Three-dimensional reconstruction of CTA showed the tumor was closely adhered to the abdominal aorta and the left internal iliac artery. The tumor was compressed and partially wrapped around the left external iliac artery. **g**: 1 year after surgery, pelvic CT showed L3–5 vertebral structurewas partially repaired, and no recurrence was seen. **h**: 1 year after surgery, CTA showed no tumor was wrapped in the left external iliac artery, and no filling defect was seen in the lumen

The clinical manifestations and imaging features of 2 patients with VMs are shown in Fig. 2 and Fig. 3. Preoperative contrast-enhanced CT showed no obvious enhancement of the mass. CTA did not show images of the thick tortuous arteriovenous, but showed cystic or cystic solid mass, with multiple phleboliths formed in the lesion. In the operation, the mass was identified as a VMs with abundant blood supply and thin tumor wall. When surgically separated, the mass was prone to rupture and massive bleeding. After operation, platelet count and coagulation function of the two VMs patients had returned to normal. Case 1 had a small amount of vein malformation remaining in the thoracic cavity and thoracic spine, was further treated with an intratumoral injection of ethanol. Two patients of KHE with KMP underwent CT scan before their surgery, which showed that the lesion was a blood-rich soft tissue tumor. Contrast-enhanced CT showed obvious tumor enhancement, and CTA scan showed abundant tumor blood supply vessels (see Fig. 4). In case 3, the area of skin defect in the lesion area was huge after tumor resection, and the skin of the tumor was used for in situ skin grafting. The skin became necrotic after operation. Stage 2 skin graft surgery was then performed once the postoperative platelet count and coagulation function were stabilized. Case 4 received post-treatment with prednisone and vincristine for 3 months. One year after the operation, the CTA showed that the bone destruction of the left attachment of L3–5 had been repaired, and the flaky soft tissue mass attached to the left pelvic wall and the left external iliac artery had been reduced.

**Fig. 5:**
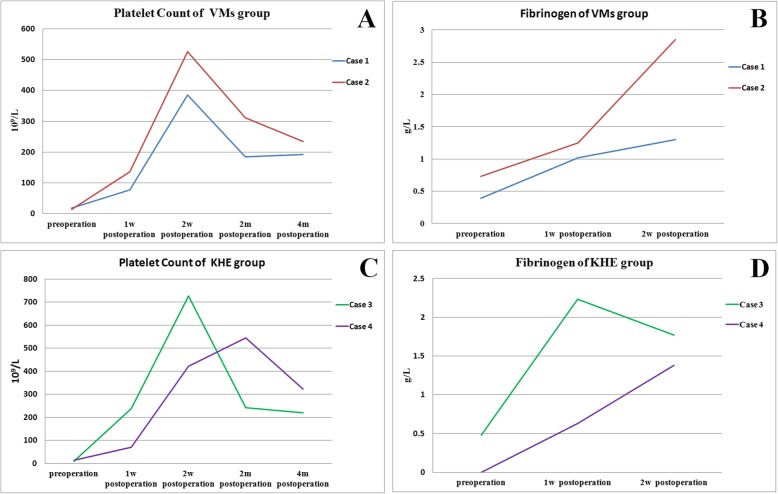
**a**: Trend of platelet changes in children before and after surgery in VMs group. **b**: Trend of fibrinogen changes in children before and after surgery in VMs group. **c**: Trend of platelet changes in children before and after surgery in KHE group. **d**: Trend of fibrinogen changes in children before and after surgery in KHE group. w: week(s), m:month(s)

The preoperation hematological metrics and surgery metrics are show in Table 2. In VMs group, the preoperative platelet count was 19 × 10^9^/L and 14 × 10^9^/L, the preoperative fibrinogen was 0.39 and 0.73 g/L, and the D-Dimer was 31.5 and 97.02 mg/L FEU. The PT, APTT, and PTINR of case 1 cannot be detected. The operation time was 385 and 195 min. The intraoperative blood loss was 1500 ml and 300 ml. The intraoperative blood transfusion was 7 U for case 1 and 1 U for case 2. In KHE group, the preoperative platelet count was 10 × 10^9^/L and 13 × 10^9^/L, the preoperative fibrinogen was 0.48 and 0 g/L, and the D-Dimer was 60.16 and 18.02 mg/L FEU. The PT, APTT, and PTINR of the 2 cases cannot be detected. The operation time was 100 and 115 min. The intraoperative blood loss for both case 3 and case 4 was 100 ml, and the intraoperative blood transfusion volume was 1 U for both cases. A comparison of platelet count and fibrinogen quantification before and after surgery is shown in Fig. 5. Platelets of the two groups returned to the normal range 1 week after surgery, then became higher than normal, and stabilized within the normal range 4 months after surgery. At 1 week postoperatively, PT, APTT, and PTINR of the two groups returned to normal levels, and fibrinogen quantitation returned to nearly normal levels 2 weeks after surgery. All patients were discharged from hospital, with no physical dysfunction, recurrence, or death. The patients were followed up for 10–26 months. There were no related postoperative complications, no tumor recurrence or progression was found, and the function of the affected limb was well recovered.

**Table 2:** Preoperation hematological metrics and surgical metrics

	VMs group		KHE group	
	Case 1	Case 2	Case 3	Case 4
Platelet count (×10^9^/L)	19	14	10	13
Fibrinogen (g/L)	0.39	0.73	0.48	0
D-Dimer (mg/L FEU)	31.5	97.02	60.16	18.02
Operation time (min)	385	195	100	115
Intraoperative blood loss (ml)	1500	300	100	100
Blood transfusion (U)	7	1	1	1

## Discussions

Infantile huge KHE with KMP or VMs, often combined with thrombocytopenia and coagulopathy, has a high mortality rate. Pharmacological management is often the first line option to achieve hemostatic stability in KMP. According to the literature, the response time of Sirolimus is 1 day to 4 weeks for KHE and 1–3 months for vascular malformations [7] [13] [18] [19]. KHE or VMs are the main lesions that cause coagulopathy and thrombocytopenia. Surgical operation of lesion resection plays an important role in the treatment of patients with thrombocytopenia and coagulopathy, but the optimal timing of surgical treatment remains controversial. Studies have shown that patients who received early surgical operation can be benefited more than patients with advanced disease. Surgery can shorten treatment time, reduce recurrence rate, and reduce drug-related side effects and complications [20]. Therefore, surgical treatment needs to be considered when the following conditions occur: 1) When the lesion is small, and the surgical removal does not cause organ damage or functional impairment; 2) when the lesion is expected to cause damage, ulcers, hemorrhage or deformity to the body [21]; 3) those that have failed medical treatment or are imminently life threatening [22]. Accurate diagnosis, complete surgical preparation, and precise and moderate resection of the lesion are key factors to the success of the procedure.

KHE can be easily misdiagnosed as local infection or inflammation, due to manifestation of red, swelling, warm, and painful mass [23] [24]. In contrast, thoracic and retroperitoneal giant vein malformation are also rare [25] [26], and easily misdiagnosed as chest wall lymphangioma or retroperitoneal malignant solid tumor. Imaging and hematology examinations are necessary before surgery. The CT performance of KHE is also different from that of VMs. The CT scan of KHE is mainly manifested as heterogeneous soft tissue mass, featured with tumors that show invasive growth, unclear boundary between the tumor and surrounding tissues, abundant blood supply vessels, and lesions that can be strengthened by enhanced scan. Most of the CT images of vascular malformations showed soft tissue density changes, and contrast-enhanced CT was characterized by uniform enhancement of soft tissue masses [27] [28]. The venous malformation was not significantly enhanced in the arterial phase. The contrast agententering the tumor showed a progressively enhanced performance; the arteriovenous malformation was also markedly enhanced early, and the thickened blood-supplying artery and dilated reflux vein were seen [29]. Common vascular malformations are easier to diagnose based on CT findings, but we need to pay attention to the special type of CT not imagined. In this study, CT findings of 2 patients with VMs showed no enhancement, and CTA also showed no images of malformed vessels within the lesion. Cho JH et al. believed that due to the lack of arterial blood vessels and capillary components, and the intratumoral vascular tortuosity, the blood flow in venous malformations was slowly retarded [30]. Slow blood stasis and thrombosis in the lesion may be the reason why the CT contrast-enhanced scan wasn’t visualized [31]. This hemodynamic change led to intratumoral thrombosis and phleboliths formation, while phlebolithsisa typical CT feature of venous malformations [30] [32].

Interventional embolization treatment has a good therapeutic effect on patients with vascular malformations complicated with thrombocytopenia and coagulopathy [33] [34] [35]. In this study, 4 patients were not treated with embolization, mainly because 2 cases of VMs did not find a clear feeding artery or vein. The other 2 KHE patients were very rich in tumor capillary network, which lacked the main blood-supplying vessels. The embolization effect is also not satisfactory. Therefore, we performed emergency surgery for patients while actively correcting the patient’s coagulation function. After infusion of fresh frozen plasma, platelets, human fibrinogen or cryoprecipitate, the patient’s coagulation function can be corrected for a short time, and immediate surgery can be considered. The large volume of the lesion, thrombocytopenia, and coagulopathy all increase the risk of surgical bleeding, intraoperative complications, and mortality. During surgery, attention should be paid to controlling the amount of bleeding, protecting important tissues and organs, and repairing defective skin. Some blood supplying arteries or veins of lesions can be blocked to reduce the amount of intraoperative blood loss (see Fig. 4c). In patients with chest wall vein malformation, the lesion base is wide and usually invading the thoracic cavity through the intercostal space. We took a method of suturing and occluding hemostasis while removing the chest wall lesion, to avoid ribs when removing the lesion, and to reserve enough tissue for suturing. Otherwise, bleeding would be difficult to control. When the thoracic vertebra was invaded by lesions, it was difficult to be completely removed, and may not be treated temporarily, in order to avoid damaging the nerve and causing dysfunction. Although the lesions in case 1 and case 4 could not be removed completely, the effect of cytoreductive surgery was also satisfactory. After the postoperative blood coagulation function is stabilized, the residual lesion can be further treated by a combination of drugs or interventional methods.

## Conclusions

For the KHE and KMP infants, we still recommend systemic medications. Intravenous sclerosis is still recommended for the treatment of VMs. However, timely and appropriate surgical treatment should be considered when it is determined that the medical treatment is ineffective or the life-threating condition has occurred before the drug become effective. A successful surgery will require precise and moderate resection of the lesion, bleeding control, and well-preserved organ functions. Our data showed that surgery can save patients’ lives when patients’ condition become critical, reduce the side effects and complications associated with drug treatment, and shorten treatment time.

## Data Availability

The used data were retrospectively retrieved from electronic medical records of the Sun Yat-Sen Memorial Hospital of Sun Yat-Sen University under the requests and approval of IRB. Further, it was claimed that the data that support the findings of this study can only be accessed by the researchers and assistants in the team. Feel free to contact the corresponding authors regarding the availability of data and materials.

## Abbreviations

APTT: Activated partial thromboplastin time
CT: Computed tomographic
CTA: Computed tomographic angiography
KMP: Kasabach-Merritt phenomenon
L3–5: Lumbar 3–5
PT: Prothrombin time
PTINR: International ratio of prothrombin
